# Efficacy and safety of bempedoic acid for prevention of cardiovascular events and diabetes: a systematic review and meta-analysis

**DOI:** 10.1186/s12933-020-01101-9

**Published:** 2020-08-12

**Authors:** Xing Wang, Yu Zhang, Huiwen Tan, Peng Wang, Xi Zha, Weelic Chong, Liangxue Zhou, Fang Fang

**Affiliations:** 1 West China Hospital, Sichuan University, No. 37, Guo Xue Xiang, Chengdu, 610041 Sichuan China; 2 Affiliated Hospital of Chengdu University, Chengdu University, Chengdu, Sichuan China; 3 Sidney Kimmel Medical College, Thomas Jefferson University, Philadelphia, PA USA

**Keywords:** Bempedoic acid, Prevention, Cardiovascular disease, Meta-analysis

## Abstract

**Background:**

Bempedoic acid is an oral, once-daily, first-in-class drug being developed for the treatment of hyperlipidemia. However, evidence of bempedoic acid use for the prevention of cardiovascular events and diabetes is lacking. Thus, we aim to evaluate the benefit and safety of bempedoic acid use for the prevention of cardiovascular events and diabetes.

**Methods:**

We searched Medline, Embase, and the Cochrane Central Register of Controlled Trials with no language restriction from inception until March 3, 2020. Pairs of reviewers independently identified randomized controlled trials comparing the use of bempedoic acid with placebo or no treatment for primary prevention of cardiovascular events in statin-intolerant patients with hypercholesterolemia. The primary outcomes were major adverse cardiac events, and percent change in LDL-C.

**Results:**

We identified 11 trials including a total of 4391 participants. Bempedoic acid use was associated with a reduction in composite cardiovascular outcome (RR 0.75, 95% CI 0.56–0.99; I^2^ = 0%). Bempedoic acid reduced LDL-C levels (MD − 22.91, 95% CI − 27.35 to − 18.47; I^2^ = 99%), and similarly reduced CRP levels (MD -24.70, 95% CI − 32.10 to − 17.30; I^2^ = 53%). Bempedoic acid was associated with a reduction in rates of new-onset or worsening diabetes (RR 0.65, 95% CI 0.44–0.96; I^2^ = 23%).

**Conclusions:**

Bempedoic acid in patients with hypercholesterolemia was associated with a lower risk of cardiovascular events and diabetes.

## Background

Cardiovascular diseases are among the principal causes of mortality, accounting for about 1 in 3 deaths in the United States [1]. Therefore, preventive interventions from the established risk factors for such diseases are a high priority. Statins are the standard of care for lower cholesterol levels and prevention of cardiovascular events. However, statin intolerance is reported to prevalent from 7 to 29%, with the predominant symptoms being muscle-related side effects [2]. Inability to tolerate statins leads to uncontrolled cholesterol levels and insufficient cardiovascular risk reduction. To reduce cardiovascular risk in these patients, the 2018 multisociety guidelines advocate the addition of non-statin agents [3].

Bempedoic acid (Esperion Therapeutics Inc, Ann Arbor, MI), as a small molecule inhibitor of ATP-citrate lyase, is an oral, once-daily, first-in-class drug being developed for the treatment of hyperlipidemia by inhibiting cholesterol synthesis [4]. Randomized clinical trials have shown the efficacy of bempedoic acid treatment on lowering low-density lipoprotein–cholesterol [5–17]. Thus, the drug has been listed in the future perspectives of 2019 ESC/EAS Guidelines for new approaches to reduce low-density lipoprotein cholesterol [18]. In 2020, the Food and Drug Administration (FDA) has approved bempedoic acid for the treatment of adults who require additional low-density lipoprotein-cholesterol lowering [19], and European Medicines Agency (EMA) has recommended approval bempedoic acid to treat adults with primary hypercholesterolemia and mixed dyslipidemia [20]. However, the benefites of bempedoic acid are limited, because current trials have not proved the potential benefits of bempedoic acid use for prevention of cardiovascular events [7, 13]. The primary criticisms of those trials have been small numbers of events, raising the probability of a type II error. We performed the systematic review and meta-analysis to evaluate the potential benefit and safety of bempedoic acid use for the prevention of cardiovascular events.

## Methods

### Protocol and guidance

The protocol of this study was registered in Open Science Framework (https://osf.io/va34s). The methods of reporting systematic review followed PRISMA guidelines [21].

### Eligibility criteria

Inclusion Criteria: Eligible studies met the following PICOS (Patients, Intervention, Comparison Outcomes, and Study design) criteria: (1) Population: statin-intolerant patients or patients that are on statins, with hypercholesterolemia (age ≥ 18); (2) Intervention: bempedoic acid; (3) Comparison intervention: placebo or no treatment; (4) Outcomes: at least one outcome of interest had to be reported. (5) Study design: randomized controlled trials.

### Outcomes

The primary outcomes were major adverse cardiac event (defined as a composite of cardiovascular death, myocardial infarction, nonfatal stroke, hospitalization for unstable angina, and coronary revascularization. Follow-up of the cardiovascular events should be at least 12 months or 48 weeks), and percent change from baseline to the respective study endpoints in low-density lipoprotein cholesterol (LDL-C). Secondary event outcomes were cardiovascular death, myocardial infarction, nonfatal stroke, hospitalization for unstable angina, coronary revascularization, percent change in C-reactive protein (CRP), and new-onset or worsening diabetes. Secondary safety outcomes were any adverse event, serious adverse event, muscular-related adverse event, decrease in glomerular filtration rate, increase in blood creatinine, increase in blood uric acid, gout, neurocognitive disorders, ALT or AST > 3 × ULN, and creatine kinase (CK) > 5 × ULN. These outcomes may be defined in individual trials with variations.

### Information sources and search strategy

We searched the electronic databases Medline, Embase, Cochrane Library of Clinical Trials from inception until March 3, 2020. Relevant clinical trial registries (ClinicalTrials.gov) were consulted regarding any ongoing studies or the availability of completed studies with reported results. We also checked the reference lists of eligible studies and screened scientific abstracts. We did not use any language or publication status restrictions. The details of the search strategy conducted are presented in Additional file 1. Table S1.

### Study selection

Two authors (YZ and XW) evaluated eligibility independently based on titles and abstracts of all reports retrieved in the electronic search. They screened the full text for potentially relevant trials when both agreed that a citation met the eligibility criteria. Discrepancies were resolved by consensus among the study team. The corresponding authors were contacted to obtain missing information and unpublished data when needed, to assess the inclusion criteria or when suitable data were not available.

### Data collection process

Data were extracted using piloted forms, independently and in duplicate by the two authors (YZ and XW). Discrepancies were resolved by consensus among the study team.

### Assessment of risk of bias and quality of evidence

Two authors (YZ and XW) examined eligible studies independently using the Cochrane risk of bias assessment tool [22]. We assessed the following domains for each study: (1) random sequence generation, (2) allocation sequence concealment, (3) blinding of participants and personnel, (4) blinding of outcome assessment, (5) completeness of outcome data, (6) selective reporting, and (7) other sources of bias. Each domain was assessed as either low, unclear, or high risk of bias. The highest risk of bias for any criteria was used to reflect the overall risk of bias for the study.

Two authors (YZ and XW) used the Grading of Recommendations, Assessment, Development and Evaluation (GRADE) approach to rate the quality of evidence and generate absolute estimates of effect for the outcomes, taking into account study limitations (risk of bias), inconsistency, imprecision, indirectness, and publication bias [23].

### Statistical analysis

The statistical analyses were performed using RevMan (5.3.3; The Cochrane Collaboration) and the meta package in R (version 3.4.3; R Project for Statistical Computing). Random-effects models were used for all outcomes. Dichotomous variables were analyzed using the Mantel–Haenszel method and were expressed as risk ratios (RR). Continuous variables were expressed as mean differences. Statistical significance testing was 2-sided and *P* < 0.05 was considered statistically significant. Heterogeneity was assessed using with the *χ*^2^ test and the I^2^ test, with I^2^ > 50% being considered substantial [24]. The possibility of publication bias was evaluated by a visual estimate of the funnel plot and by the regression tests Egger test, Begg test, and Harbord test when ten or more trials were pooled [25]. Analyses for all outcomes were done on an intention-to-treat basis.

### Sensitivity analyses

Sensitivity analyses were conducted for the primary outcome by (1) excluding trials with unclear or higher risk of bias (2) using inverse variance method; and (3) using fixed-effect models.

### Subgroup analysis

We planned subgroup analyses for major adverse cardiac event and percent change in LDL-C for the following variables: (1) whether patients with a history of statin intolerance; (2) whether treatment with a combination of bempedoic acid and ezetimibe; (3) whether treatment with a combination of bempedoic acid and maximally tolerated statin therapy; (4) whether treatment with stains as background therapy.

## Results

### Description of included studies

**Fig. 1:**
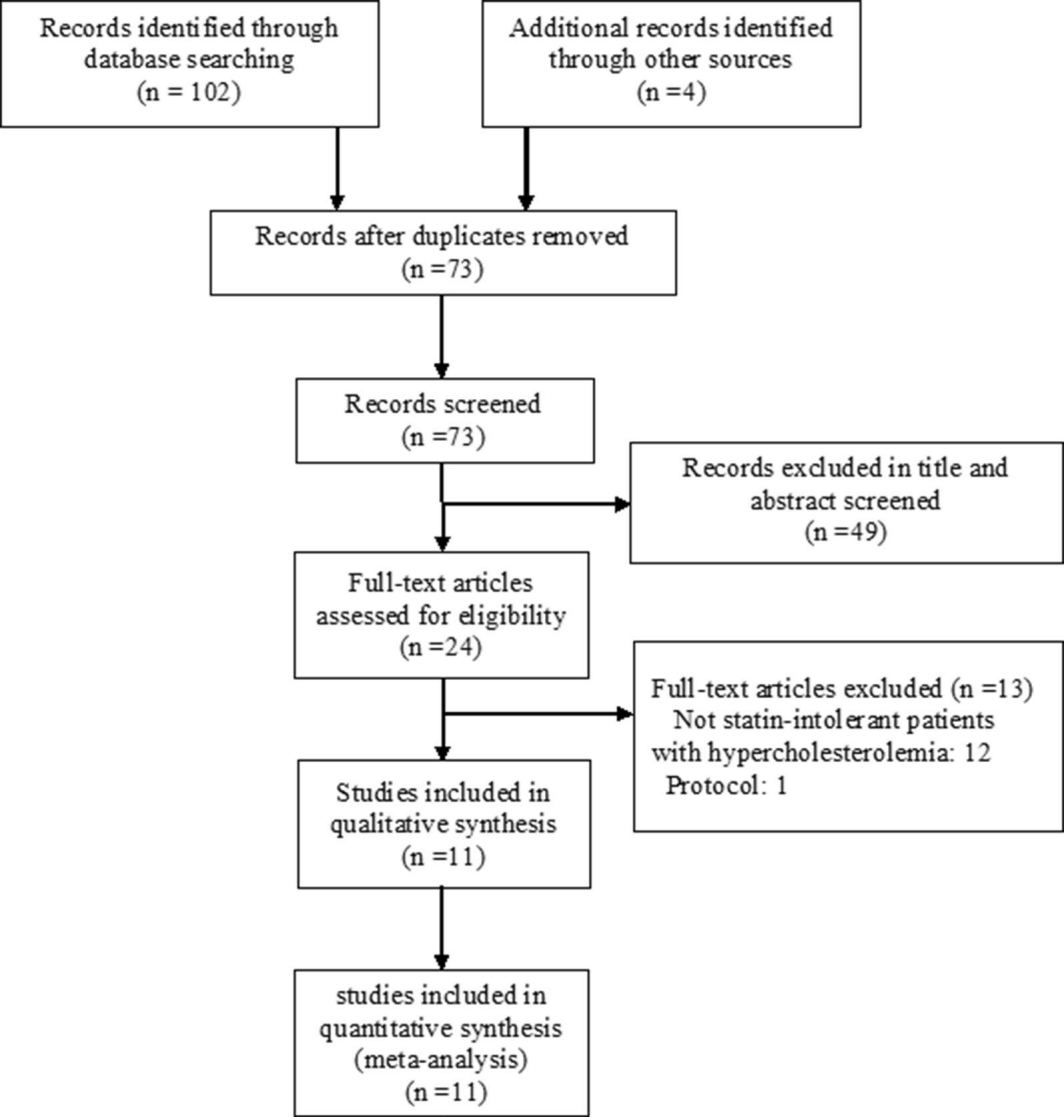
Flow diagram showing final included and excluded studies

We identified 106 records. After screening, eleven trials [5–15] with a total of 4391 participants met our inclusion criteria. Figure 1 details the study selection process. Descriptive details of the eligible trials are presented in Table 1. Study sizes ranged from 56 to 2230 participants; the mean age ranged from 53.3 to 64.7 years; the percentage of female ranged from 26.1 to 63.6%. Two trials [7, 13] have a follow-up period of at least 1 year, whereas nine trial [5, 6, 8–12, 14, 15] have follow-up periods less than 1 year.

**Table 1:** Characteristics of studies included in the systematic review

Trial	Country	Patients, n	Female (%)	Age, years	BMI, kg/m^2^	Follow up (weeks)	Baseline for LDL-C(mg/dl)	Characteristics of patients	Dose of bempedoic acid
Ballantyne 2013	US	177	44.6	57.5	28.2	12	166.2	No stain used	40, 80, or 120 mg daily
Gutierrez 2014	US	60	38.3	55.7	29.9	12	126.8	No stain used	180 mg daily
Thompson 2015	US	56	50.0	63.5	29.7	12	179.05	Statin intolerant	120 mg, 180 mg, or 240 mg
Ballantyne 2016	US	133	59.4	57.3	30.3	12	135.6	Stable statin background therapy	180 mg daily
Thompson 2016	US	149	71.1	59.7	29.7	12	163.9	With or without statin intolerant	120 mg or 180 mg
Ballantyne 2018	US	269	61.3	63.8	29.8	12	127.6	Statin intolerance	180 mg daily
Laufs 2019	Germany	345	50.4	65.2	30.3	24	157.6	Statin intolerance	180 mg daily
Ray 2019	UK	2230	27.0	66.1	NR	52	103.2	Maximally tolerated statin therapy	180 mg daily
Ballantyne 2019	US	129	50.4	65.1	30.6	12	148.08	Maximally tolerated statin therapy	180 mg daily
Goldberg 2019	US	779	36.3	64.3	30.2	52	120.4	Maximally tolerated statin therapy	180 mg daily
Lalwani 2019	US	64	48.4	58.0	31.0	4	76.4	High-intensity statin background therapy	180 mg daily

*NR* not reported, *BMI* body-mass index, *LDL-* low-density lipoprotein cholesterol

### Risk of bias and quality of evidence

The overall quality of the 11 included trials was moderate (Figure S1 and S2 in the Additional file 1.); 6 trials [6, 7, 10, 13–15] had low risk of bias, 1 trial [8] had unclear risk of bias, and 4 trials [5, 9, 11, 12] had high risk of bias. Table 2 shows a summary of findings for all outcomes. The quality of evidence assessed with the GRADE approach was high for major adverse cardiac events.

**Table 2:** Summary of findings and strength of evidence in studies

Outcome	No. of patients (Trials)	RR/MD (95% CI)	I^2^	Absolute effect estimates (per 1000)			Quality of the evidence
				Intervention	Control	Difference	
Cardiovascular events	3008 (2)	0.75 [0.56, 0.99]	0%	56	74	− 19 [− 1, − 33]	High
Percent change of LDL-C	3957 (9)	− 22.91 [− 27.35, − 18.47]	99%	–	–	–	Moderate^#^
New-onset or worsening diabetes	3621 (4)	0.65 [0.44, 0.96]	23%	38	58	− 20 [− 2, − 32]	Moderate^§^
Percent change of CRP	3555 (7)	− 24.70 [− 32.10, − 17.30]	53%	–	–	–	Moderate^#^
Myocardial infarction	3008 (2)	0.54 [0.25, 1.15]	37%	12	22	− 10 [− 17, 3]	Moderate^§,#^
Coronary revascularization	3008 (2)	0.74 [0.50, 1.10]	0%	29	39	− 10 [− 20, 4]	Moderate^§^
Cardiovascular death	3008 (2)	1.65 [0.46, 5.98]	0%	5	3	2 [− 2, 15]	Moderate^§^
Nonfatal stroke	3008 (2)	1.11 [0.34, 3.61]	0%	4	4	0 [− 3, 10]	Moderate^§^
Hospitalization for unstable angina	2574 (2)	0.84 [0.41, 1.73]	51%	11	13	− 2[− 8, 9]	Low^#,§^
Any adverse event	4188 (9)	1.01 [0.97, 1.05]	40%	712	705	7 [− 21, 35]	Low^&,^^#^
Serious adverse event	4184 (9)	1.06 [0.89, 1.26]	0%	126	119	7 [− 13, 31]	Moderate^&^
Muscular-related adverse event	2703 (3)	1.12 [0.80, 1.56]	26%	120	107	13 [− 21, 60]	Moderate^§^
Decrease in glomerular filtration rate	3276 (3)	3.61 [0.81, 16.04]	0%	4	1	3 [− 1, 14]	Low*
Increase in blood creatinine	3482 (4)	2.15[0.81, 5.69]	0%	6	3	3 [− 1, 16]	Moderate^§^
Increase in blood uric acid	1176 (3)	3.76 [1.24, 11.39]	0%	30	8	22 [2,81]	Low*
Gout	3421 (4)	2.37 [0.88, 6.36]	12%	12	5	7 [− 1, 43]	Moderate^§^
Neurocognitive disorders	3076 (3)	0.94 [0.41, 2.17]	0%	8	8	0 [− 5, 9]	Moderate^§^
ALT or AST > 3 × ULN	3382 (5)	1.97 [0.61, 6.34]	0%	6	3	3 [− 1, 14]	Moderate^§^
CK > 5 × ULN	3382 (5)	1.31 [0.23, 7.50]	19%	3	2	1 [− 1, 12]	Moderate^§^

*LDL-C* Low density lipoprotein cholesterol, *CRP* C-reactive protein, *CK* Creatine kinase, *ALT* Alanine aminotransferase, *AST* Aspartate aminotransferase, *ULN* Upper limit of the normal range, *RR* Risk ratio, *MD* Mean difference

^&^Publication bias

^#^Inconsistency

^§^Imprecision

*Very serious imprecision

### Cardiovascular events

**Fig. 2:**
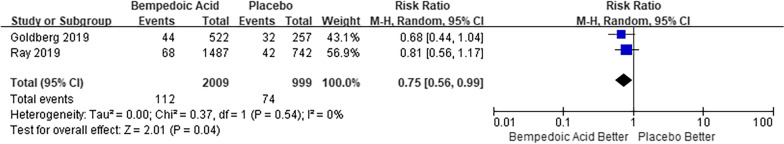
Random-effects meta-analysis of bempedoic acid on composite cardiovascular outcome

There were 2 trials [7, 13], including a total of 3008 participants with data available regarding major adverse cardiovascular events. Specifically, we performed a composite tally of cardiovascular death, myocardial infarction, nonfatal stroke, hospitalization for unstable angina, and coronary revascularization. The use of bempedoic acid was associated with reductions in the composite cardiovascular outcome (RR 0.75, 95% CI 0.56–0.99; I^2^ = 0%; Fig. 2). Other cardiovascular events were reported in Table 2. The use of bempedoic acid was not associated with reductions in cardiovascular death, myocardial infarction, nonfatal stroke, hospitalization for unstable angina, and coronary revascularization, compared individually with placebo (Additional file 1. Figure S3–S7). Subgroup analysis for major adverse cardiovascular events did not detect any beneficial effect in any specific subgroups (Additional file 1. Figure S8).

### Percent change in LDL-C and CRP

**Fig. 3:**
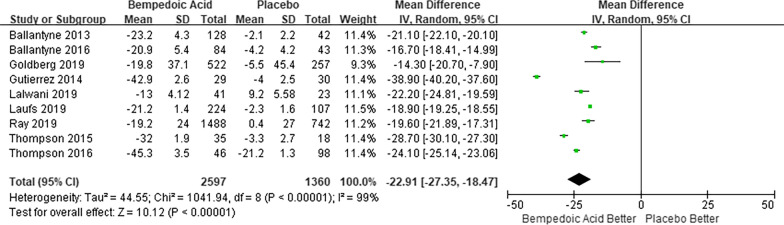
Random-effects meta-analysis of bempedoic acid on percent change in LDL-C. *LDL-C* Low density lipoprotein cholesterol

There were 9 trials [6, 8, 12, 26–31], including a total of 3957 participants with data available regarding the percent change in LDL-C from baseline to the respective study endpoints. The pooled results showed that using bempedoic acid resulted in lower LDL-C compared with placebo, with a mean difference of 22.91% (95% CI − 27.35 to − 18.47%; I^2^ = 99%; Fig. 3). Subgroup analysis for percent change in LDL-C revealed subgroup of no stains using as background therapy benefit more on LDL-C lowering (P = 0.03; Additional file Figure S9). Seven trials including 3555 participants reported percent change in CRP. The pooled results showed that bempedoic acid reduced CRP levels compared with placebo, with a mean difference of 24.70% (95% CI − 32.10 to − 17.30%; I^2^ = 53%; Additional file 1. Figure S10).

### New-onset or worsening diabetes

**Fig. 4:**
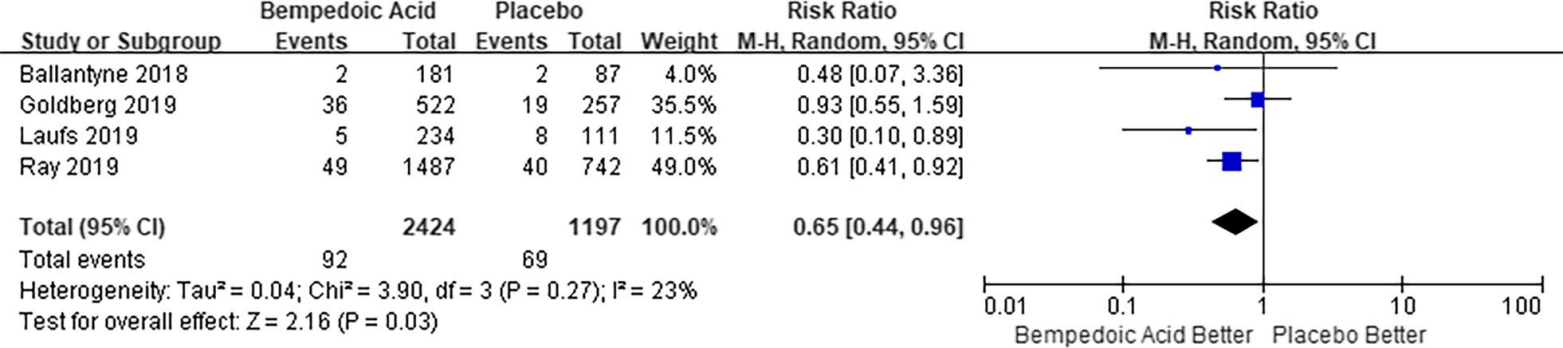
Random-effects meta-analysis of bempedoic acid on new-onset or worsening diabetes

For the outcome of new-onset or worsening diabetes, four studies [7, 10, 11, 13] reported a total of 161 events [92/2424 (3.7%) with bempedoic acid and 69/1197 (5.7%) with placebo]. The use of bempedoic acid was associated with a reduction in new-onset or worsening diabetes (RR 0.65, 95% CI 0.44–0.96; I^2^ = 23%; Fig. 4).

### Safety outcomes

Table 2 summarized findings of safety outcomes. Bempedoic acid was associated with elevated levels of blood uric acid (Additional file 1. Figure S11), but not associated with a decrease in glomerular filtration rate. Bempedoic acid was not associated with an increased incidence of any adverse event, serious adverse event or muscle-related adverse event. Also, bempedoic acid was not associated with increased blood creatinine, gout, neurocognitive disorders, incidence of ALT or AST > 3 × ULN, or incidence of creatine kinase (CK) > 5 × ULN (Additional file 1. Figure S12–S20).

### Sensitivity analysis

Similar results were observed for the composite cardiovascular outcome in all conducted sensitivity analyses by excluding trials with unclear or higher risk of bias, using inverse variance method, and using fixed-effect models (Additional file 1. Table S2).

## Discussion

In this meta-analysis of 11 RCTs enrolling 4391 patients, the use of bempedoic acid was associated with reductions in the composite cardiovascular outcome consisting of cardiovascular death, myocardial infarction, nonfatal stroke, hospitalization for unstable angina, and coronary revascularization in statin-intolerant patients with hypercholesterolemia. Moreover, bempedoic acid use was associated with a reduced risk of diabetes.

### Comparison with existing data

To the best of our knowledge, this study is the first meta-analysis assessing the effect of bempedoic acid on cardiovascular events. The previous meta-analysis has evaluated the use of bempedoic acid to reduce LDL cholesterol [17], and had analyzed 5 trials with a total of 625 patients with hypercholesterolemia, concluding that bempedoic acid led to significantly lower LDL cholesterol levels without adverse events. The previous review did not assess the efficacy of bempedoic acid on cardiovascular events and may be underpowered to assess adverse drug events due to the small sample size [17].

This study builds on the previous meta-analysis mainly through the inclusion of two large trials [7, 13], the CLEAR Harmony trial [13] and the CLEAR Wisdom trial [7]. However, both trials were not designed to assess the effect on cardiovascular events, and thus, individually did not observe significant between-group differences in the incidence of cardiovascular events.

Another important finding of our analysis is that bempedoic acid was associated with a reduction in new-onset or worsening diabetes. This phenomenon may be related to AMP-activated protein kinase (AMPK), since bempedoic acid plays a dual role in both activation of hepatic AMPK signaling pathway and inhibitory activity against hepatic ATP-citrate lyase (ACL) [32, 33].

## Strengths and limitations

The major strength in our review is the strict methodology implemented which followed the recommendations of the Cochrane Collaboration and PRISMA statement, including a protocol, an up-to-date literature search and study selection, data extraction and risk of bias assessment by two independent investigators. We also included GRADE to assess the degree of certainty in pooled estimates of effect and presented absolute and relative risks.

This study has several limitations. First, the cardiovascular outcomes might be imprecise, seen as a wider CI around the estimate of the effect, due to the relatively few patients included. We have downgraded the quality of evidence of these outcomes due to imprecision of the individual studies.

Second, there is significant clinical heterogeneity in the bempedoic acid dose utilized, duration of treatment, and outcome definitions across the trials included in this meta-analysis.

Third, only 2 trials reported data with 1 year of follow-up, but other trials had less. More trials with longer follow-up are required to examine whether the benefits of bempedoic acid are enhanced over time and whether bempedoic acid can ultimately lower the rate of mortality.

Fourth, the small number of trials included to analyze the major adverse cardiac events led to the inability to detect the presence of publication bias. However, the risk of publication bias was low because all trials individually had negative results in the major adverse cardiac events.

Fifth, heterogeneity might exist in the outcome of new-onset or worsening diabetes. The two largest trials varied in the effect point of new-onset or worsening diabetes (RR 0.93 vs RR 0.61), with some statistical heterogeneity (I^2^ = 23%). We have downgraded the quality of evidence of new-onset or worsening diabetes because of inconsistencies.

### Future research and other pharmacotherapies

The results of the present meta-analysis indicate that more clinical trials are warranted to investigate remaining questions about the potentially beneficial effect of bempedoic acid on cardiovascular outcomes. Major variations in the study protocols of these trials included in the meta-analysis indicate that the optimal dosing of bempedoic acid remains uncertain. Likewise, it is uncertain if there are subgroups of patients who are more likely to benefit from bempedoic acid. Moreover, longer follow-ups could help provide much-needed data on the effectiveness on cardiovascular outcomes, long-term safety, and tolerability of bempedoic acid.

In addition to bempedoic acid [34, 35], there are many novel therapeutic drugs that have demonstrated the ability to lower LDL-C levels or further reduce the risk of major cardiovascular events in both non‑diabetic patients and diabetic patients [36–38]. These drugs span a variety of mechanisms, such as proprotein convertase subtilisin/kexin type 9 (PCSK9) inhibitor [39, 40], peroxisome proliferators-activated receptor (PPAR) inhibitor [41, 42], and IL-1β inhibitor [43]. Many of the novel therapeutic agents are undergoing clinical evaluation, and some of them have already approved by the FDA. The use of non-statin therapies for primary prevention of hyperlipidemia in subpopulations of patients who do not tolerate statins remain an area of active investigation, and clinicians should still refer to existing guidelines for initial pharmacotherapeutic selection in patients with hyperlipidemia.

## Conclusions

The use of bempedoic acid in patients with hypercholesterolemia was associated with a lower risk of cardiovascular events and DM. Morevover, bempedoic acid resulted in a significant lowering of LDL-C level and CRP level. More trials with longer follow-ups are needed to confirm the overall result and identify subgroups that benefit the most from the use of bempedoic acid.

## Supplementary information

**Additional file 1: Table S1.** Search Strategy. **Table S2.** Sensitivity analyses. **Figure S1.** Risk of bias summary. **Figure S2.** Risk of bias graph. **Figure S3.** Forest plot for bempedoic acid on cardiovascular death. **Figure S4.** Forest plot for bempedoic acid on nonfatal stroke. **Figure S5.** Forest plot for bempedoic acid on myocardial infarction. **Figure S6.** Forest plot for bempedoic acid on coronary revascularization. **Figure S7.** Forest plot for bempedoic acid on hospitalization for unstable angina. **Figure S8.** Subgroup analyses of composite cardiovascular outcome. **Figure S9.** Subgroup analyses of percent change of LDL-C. **Figure S10.** Forest plot for bempedoic acid on percent change of CRP. **Figure S11.** Forest plot for bempedoic acid on blood uric acid. **Figure S12.** Forest plot for bempedoic acid on any adverse events. **Figure S13.** Forest plot for bempedoic acid on serious adverse event. **Figure S14.** Forest plot for bempedoic acid on muscular-related adverse event. **Figure S15.** Forest plot for bempedoic acid on ALT or AST >3× ULN. **Figure S16.** Forest plot for bempedoic acid on creatine kinase (CK) >5× ULN. **Figure S17.** Forest plot for bempedoic acid on glomerular filtration rate. **Figure S18.** Forest plot for bempedoic acid on blood creatinine. **Figure S19.** Forest plot for bempedoic acid on gout. **Figure S20.** Forest plot for bempedoic acid on neurocognitive disorders.

## Data Availability

Not applicable.

## Abbreviations

BMI: Body mass index
CI: Confidence interval
DM: diabetes mellitus
GRADE: Recommendations, assessment, development and evaluation
LDL-C: Low density lipoprotein cholesterol
CRP: C-reactive protein
ALT: Alanine aminotransferase
AST: Aspartate aminotransferase
ULN: Upper limit of the normal range
RR: Risk ratio
