# Supplementary material for: Efficacy and safety of bempedoic acid for prevention of cardiovascular events and diabetes: a systematic review and meta-analysis
Source: Cardiovasc Diabetol. 2020 Aug 12;19:128. doi: 10.1186/s12933-020-01101-9 (PMC7425167; doi:10.1186/s12933-020-01101-9)

#### Supplemental e-material

| Table S1: Search Strategy: |
| --- |
| Table S2: Sensitivity analyses |
| Figure S1: Risk of bias summary |
| Figure S2: Risk of bias graph |
| Figure S3 Forest plot for bempedoic acid on cardiovascular death |
| Figure S4: Forest plot for bempedoic acid on nonfatal stroke |
| Figure S5: Forest plot for bempedoic acid on myocardial infarction |
| Figure S6: Forest plot for bempedoic acid on coronary revascularization |
| Figure S7: Forest plot for bempedoic acid on hospitalization for unstable angina |
| Figure S8: Subgroup analyses of composite cardiovascular outcome |
| Figure S9: Subgroup analyses of percent change of LDL-C |
| Figure S10: Forest plot for bempedoic acid on percent change of CRP |
| Figure S11: Forest plot for bempedoic acid on blood uric acid |
| Figure S12: Forest plot for bempedoic acid on any adverse events |
| Figure S13: Forest plot for bempedoic acid on serious adverse event |
| Figure S14: Forest plot for bempedoic acid on muscular-related adverse event |
| Figure S15: Forest plot for bempedoic acid on ALT or AST >3× ULN |
| Figure S16: Forest plot for bempedoic acid on creatine kinase (CK) >5× ULN |
| Figure S17: Forest plot for bempedoic acid on glomerular filtration rate |
| Figure S18: Forest plot for bempedoic acid on blood creatinine |
| Figure S19: Forest plot for bempedoic acid on gout |
| Figure S20: Forest plot for bempedoic acid on neurocognitive disorders |

##### Table S1: Search Strategy:

| **EMBASE** | | |
| --- | --- | --- |
| 1 | exp bempedoic acid/ | 127 |
| 2 | ETC-1002: ab,kw,ti | 42 |
| 3 | #1 or #2 | 146 |
| 4 | exp hypercholesterolemia/ or exp familial hypercholesterolemia/ | 69516 |
| 5 | high cholesterol levels.ab,kw,ti. | 757 |
| 6 | hypercholesterolemic.ab,kw,ti. | 8813 |
| 7 | elevated cholesterol:ab,kw,ti | 1709 |
| 8 | Hyperlipidemia:ab,kw,ti | 38235 |
| 9 | dyslipidemia:ab,kw,ti | 49513 |
| 10 | #4 or #5 or #6 or #7 or #8 or #9 | 152413 |
| 11 | exp randomized controlled trial/ | 594223 |
| 12 | 'trial*':ab,kw,ti OR 'random*':ab,kw,ti OR 'blind*':ab,kw,ti | 2481762 |
| 13 | 11 or 12 | 2557116 |
| 14 | 3 and 10 and 13 | 60 |
| 15 | exp human/ |  |
| 16 | 14 and 15 | 62 |
| **OVID** | | |
| 1 | bempedoic acid. ab,kw,ti | 38 |
| 2 | (ETC-1002 or ESP55016 or ESP-55016).ab,kw,ti. | 25 |
| 3 | #1 or #2 | 50 |
| 4 | exp hypercholesterolemia/ or exp familial hypercholesterolemia/ | 31814 |
| 5 | high cholesterol levels.ab,kw,ti. | 548 |
| 6 | hypercholesterolemic.ab,kw,ti. | 6805 |
| 7 | elevated cholesterol.ab,kw,ti | 1226 |
| 8 | Hyperlipidemia.ab,kw,ti | 22195 |
| 9 | Dyslipidemia.ab,kw,ti | 27025 |
| 10 | #4 or #5 or #6 or #7 or #8 or #9 | 80738 |
| 11 | exp randomized controlled trial/ |  |
| 12 | 'trial*'.ab,kw,ti OR 'random*'.ab,kw,ti OR 'blind*'.ab,kw,ti |  |
| 13 | 11 or 12 | 1882958 |
| 14 | 3 and 10 and 13 | 19 |
| 15 | exp human/ |  |
| 16 | 14 and 15 | 17 |
| **CENTRAL** | | |
| 1 | bempedoic acid: ab,kw,ti | 37 |
| 2 | ETC-1002: ab,kw,ti | 648 |
| 3 | #1 or #2 | 666 |
| 4 | exp hypercholesterolemia/ or exp familial hypercholesterolemia/ | 3783 |
| 5 | high cholesterol levels.ab,kw,ti. | 76 |
| 6 | hypercholesterolemic.ab,kw,ti. | 1686 |
| 7 | elevated cholesterol:ab,kw,ti | 222 |
| 8 | Hyperlipidemia:ab,kw,ti | 4070 |
| 9 | dyslipidemia:ab,kw,ti | 4746 |
| 10 | #4 or #5 or #6 or #7 or #8 or #9 | 12797 |
| 11 | exp randomized controlled trial/ |  |
| 12 | 'trial*':ab,kw,ti OR 'random*':ab,kw,ti OR 'blind*':ab,kw,ti |  |
| 13 | 11 or 12 | 1167639 |
| 14 | 3 and 10 and 13 | 23 |

##### Table S2: Sensitivity analyses

|  | Cardiovascular events | | New-onset or worsening diabetes | | Percent change in LDL-C | |
| --- | --- | --- | --- | --- | --- | --- |
|  | RR, 95% CI | I^2^ | RR, 95% CI | I^2^ | MD, 95%CI | I^2^ |
| excluding trials with unclear or higher risk of bias | 0.75[0.56, 0.99] | 0% | 0.71[0.51, 0.97] | 0% | -24.65 [-25.39, -23.90] | 94% |
| using inverse variance method | 0.75[0.56, 0.99] | 0% | 0.65[0.44, 0.96] | 23% | — | — |
| using fixed-effect models | 0.75[0.57, 1.00] | 0% | 0.66[0.49, 0.89] | 23% | -20.91 [-21.21, -20.62] | 99% |

### **Figure S1: Risk of bias summary: review authors' judgements about each risk of bias item for each included study.**


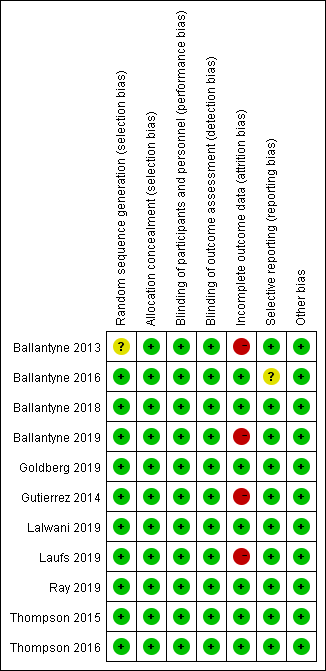


### **Figure S2: Risk of bias graph: review authors' judgements about each risk of bias item presented as percentages across all included studies**


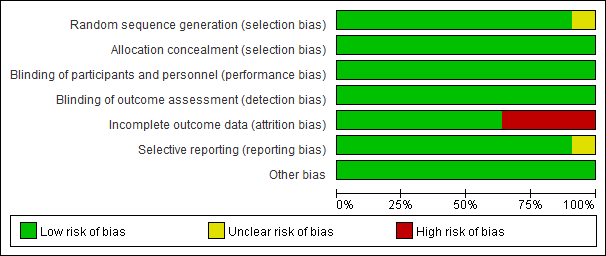


##### Figure S3 Forest plot for bempedoic acid on death from cardiovascular death


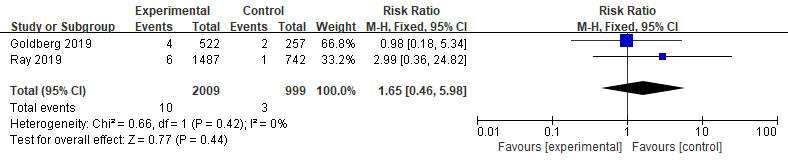


##### Figure S4: Forest plot for bempedoic acid on nonfatal stroke

###
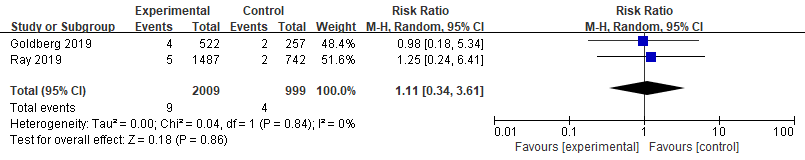


##### Figure S5: Forest plot for bempedoic acid on myocardial infarction
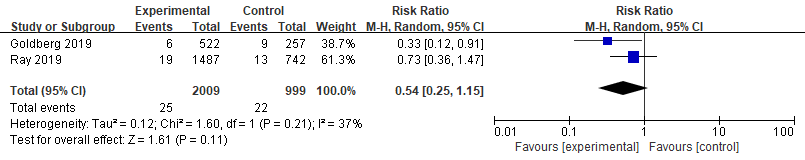


##### Figure S6: Forest plot for bempedoic acid on coronary revascularization


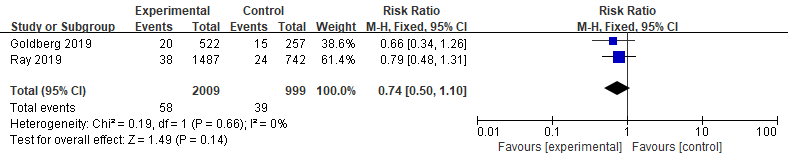


##### Figure S7: Forest plot for bempedoic acid on hospitalization for unstable angina


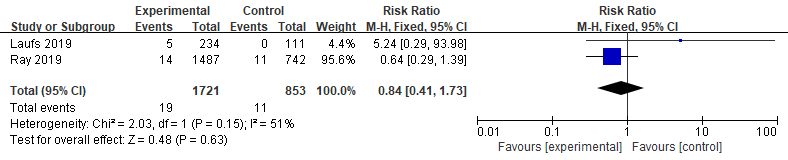


##### Figure S8: Subgroup analyses of composite cardiovascular outcome


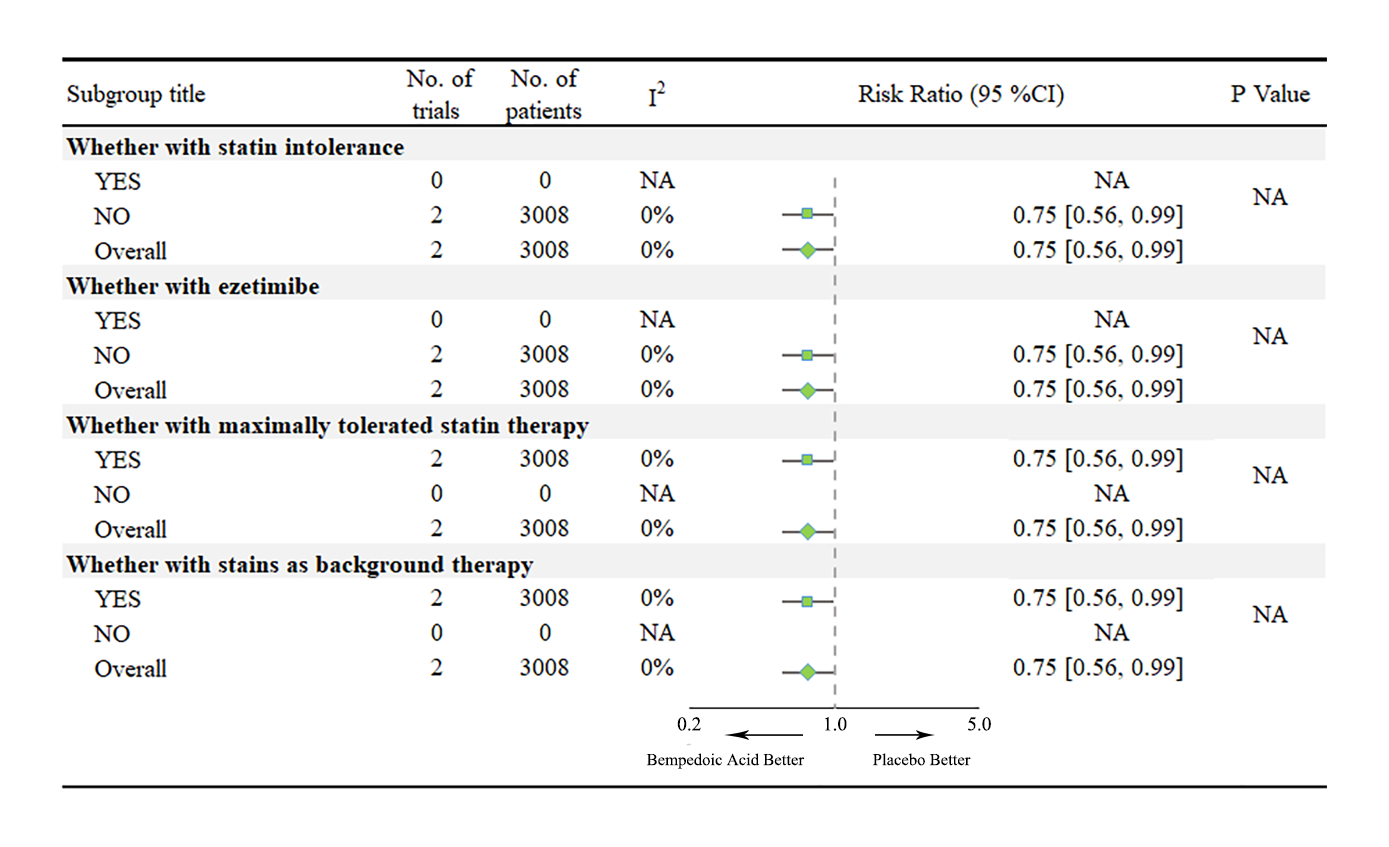


##### Figure S9: Subgroup analyses of percent change of LDL-C over time


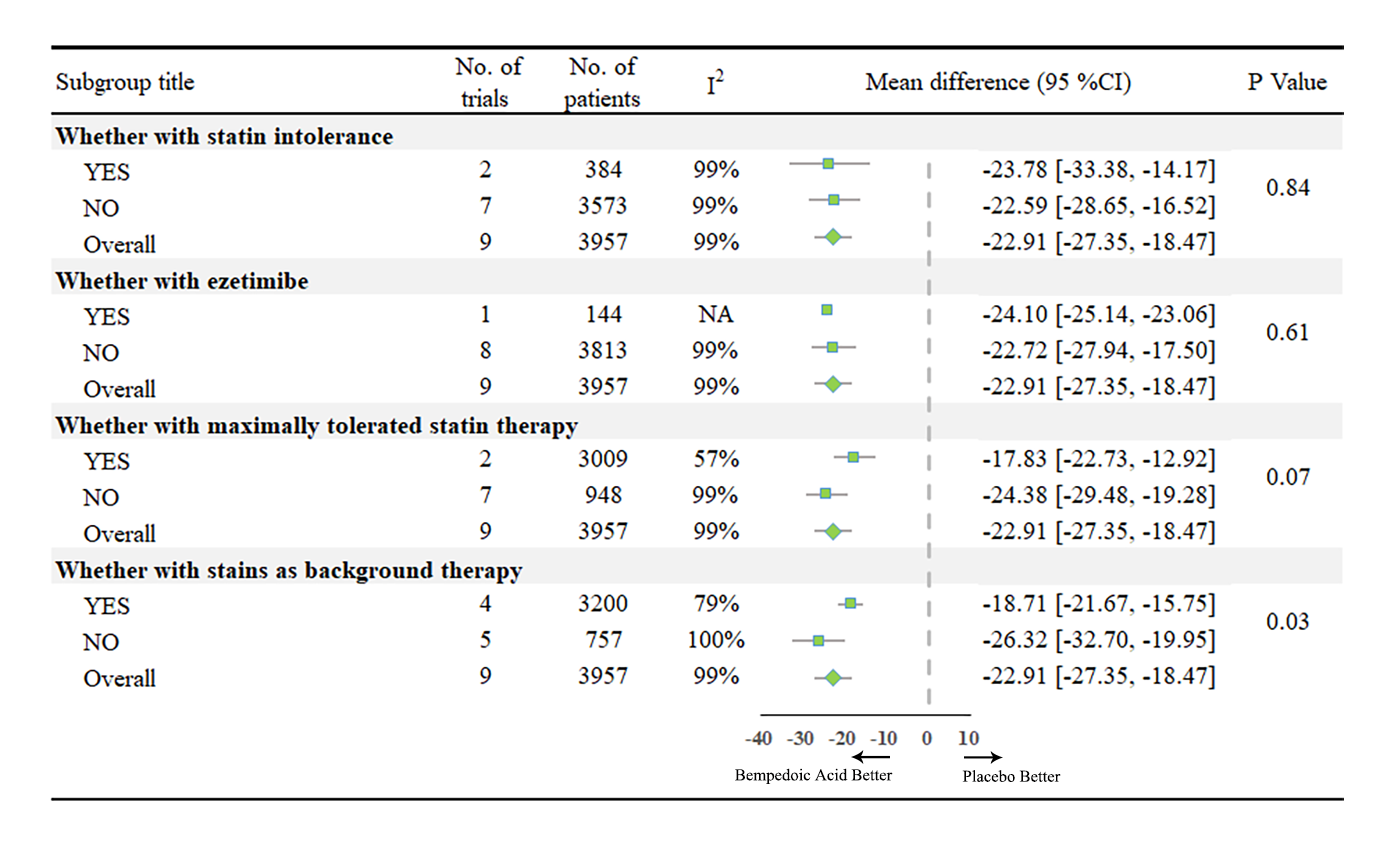


##### Figure S10: Forest plot for bempedoic acid on percent change of CRP over time

#####
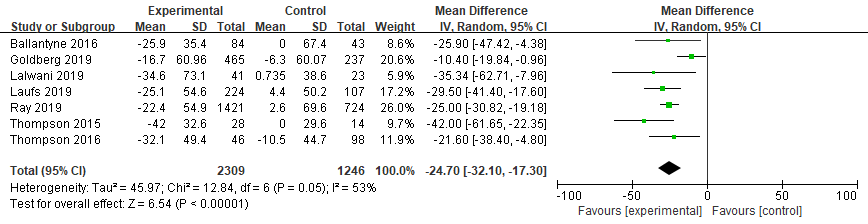


##### Figure S11: Forest plot for bempedoic acid on blood uric acid. RR > 1 indicates that blood uric acid is higher in the patients treated with bempedoic acid

#####
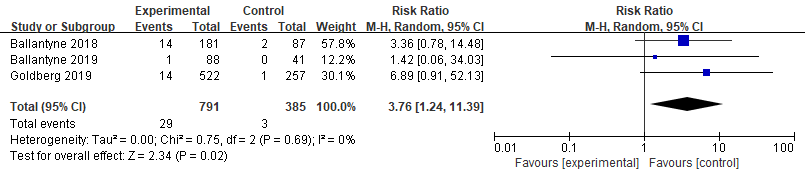


##### Figure S12: Forest plot for bempedoic acid on any adverse events


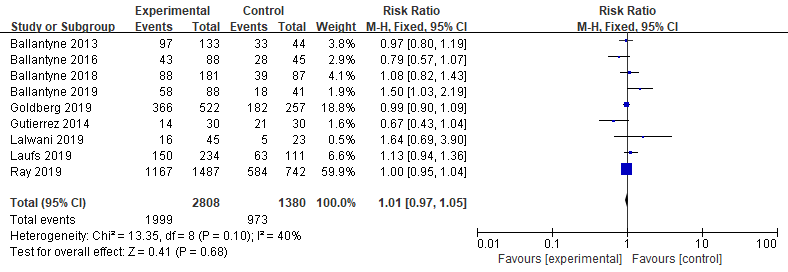


#####

##### Figure S13: Forest plot for bempedoic acid on serious adverse event


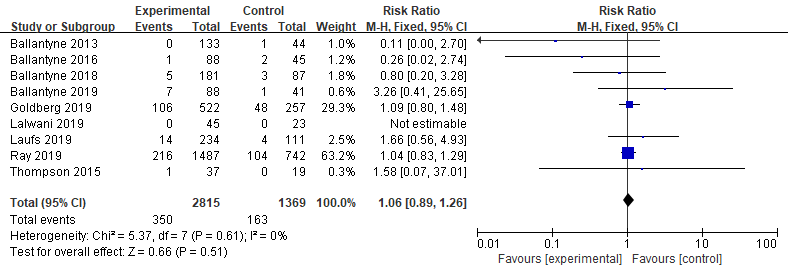


##### Figure S14: Forest plot for bempedoic acid on muscular related-adverse event


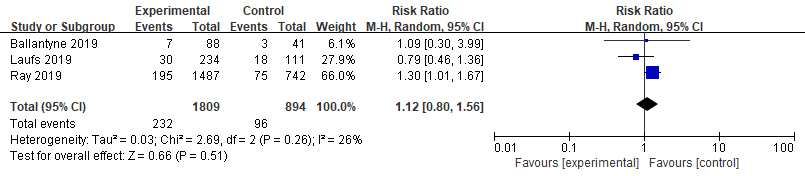


##### Figure S15: Forest plot for bempedoic acid on ALT or AST >3× ULN


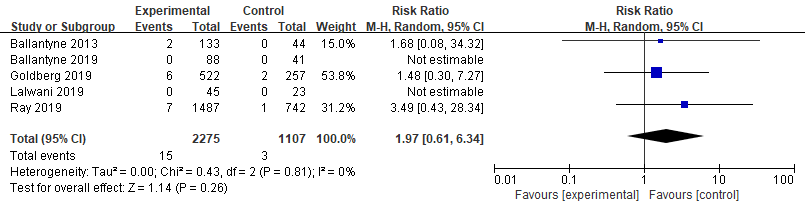


##### Figure S16: Forest plot for bempedoic acid on creatine kinase (CK) >5× ULN


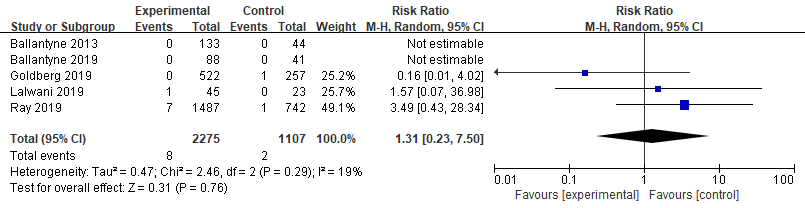


##### Figure S17: Forest plot for bempedoic acid on glomerular filtration rate


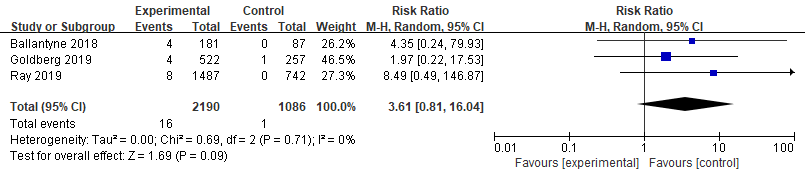


##### Figure S18: Forest plot for bempedoic acid on blood creatinine


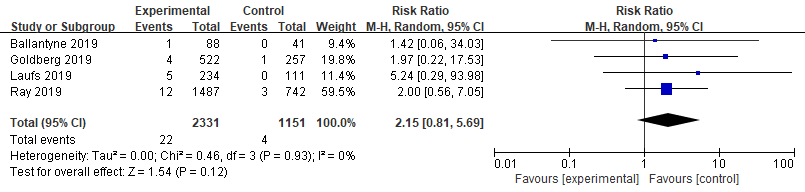


##### Figure S19: Forest plot for bempedoic acid on gout


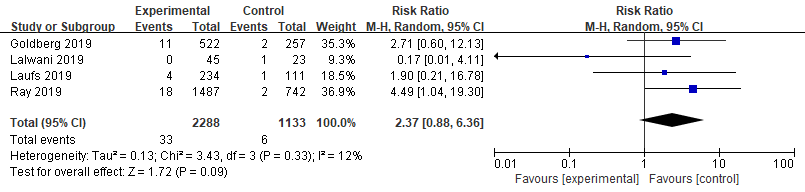


##### Figure S20: Forest plot for bempedoic acid on neurocognitive disorders


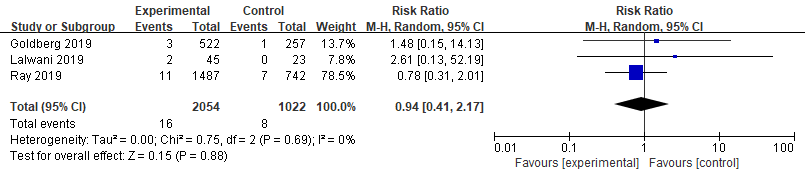

Supplement: Supplementary file 1 — Additional file 1: Table S1. Search Strategy. Table S2. Sensitivity analyses. Figure S1. Risk of bias summary. Figure S2. Risk of bias graph. Figure S3. Forest plot for bempedoic acid on cardiovascular death. Figure S4. Forest plot for bempedoic acid on nonfatal stroke. Figure S5. Forest plot for bempedoic acid on myocardial infarction. Figure S6. Forest plot for bempedoic acid on coronary revascularization. Figure S7. Forest plot for bempedoic acid on hospitalization for unstable angina. Figure S8. Subgroup analyses of composite cardiovascular outcome. Figure S9. Subgroup analyses of percent change of LDL-C. Figure S10. Forest plot for bempedoic acid on percent change of CRP. Figure S11. Forest plot for bempedoic acid on blood uric acid. Figure S12. Forest plot for bempedoic acid on any adverse events. Figure S13. Forest plot for bempedoic acid on serious adverse event. Figure S14. Forest plot for bempedoic acid on muscular-related adverse event. Figure S15. Forest plot for bempedoic acid on ALT or AST >3× ULN. Figure S16. Forest plot for bempedoic acid on creatine kinase (CK) >5× ULN. Figure S17. Forest plot for bempedoic acid on glomerular filtration rate. Figure S18. Forest plot for bempedoic acid on blood creatinine. Figure S19. Forest plot for bempedoic acid on gout. Figure S20. Forest plot for bempedoic acid on neurocognitive disorders. [file 12933_2020_1101_MOESM1_ESM.docx]
